# Revisiting Bivalve Mollusk Myogenesis: New Immunocytochemical Evidence from *Magallana gigas*

**DOI:** 10.3390/cells15211976

**Published:** 2026-10-29

**Authors:** Ilya Vyatchin, Vyacheslav Dyachuk

**Affiliations:** Laboratory of Cell Biophysics, A.V. Zhirmunsky National Scientific Center of Marine Biology, Far Eastern Branch of the Russian Academy of Sciences, Vladivostok 690041, Russia

**Keywords:** muscle proteins, catch, development, myofibrillogenesis, myogenesis, larvae, striated and smooth muscle

## Abstract

This study investigated the formation of the contractile apparatus in bivalve mollusk larvae. Elucidation of the molecular aspects of larval development in bivalves is significantly complicated by the lack of antibodies that can selectively label proteins and their structures. To address this issue, polyclonal antibodies were produced against main proteins of bivalve muscles. Immunohistochemistry was used to obtain high-quality confocal images of muscles in bivalve larvae to describe the inclusion of main muscle proteins in the contractile apparatus during the development of the oyster *Magallana gigas*. The appearance of these proteins in muscle cells was not uniform and, surprisingly, the leading protein in the formation of muscle structures, including striations, was twitchin. The findings obtained support the hypothesis that this protein, similar to vertebrate titin (a protein from the same family), may play an essential role in the assembly of the contractile apparatus. Like another protein detected during this study, myorod, twitchin is believed to be involved in the formation of the catch state (an energy-efficient state of molluskan smooth muscles). The strong evidence for the presence of these proteins within the striated muscles in the larval stage of bivalves raises questions about the origin of this important evolutionary adaptation.

## 1. Introduction

Bivalve mollusks are a diverse class of animals comprising approximately 15,000 species. Despite this great diversity, they share one common feature: all bivalves studied in this regard possess muscles that can enter the “catch” state [1]. This state is specific to the retractor [2] and smooth adductor [3] muscles of adult bivalves, which allows them to maintain tension for extended periods with almost no energy expenditure [4,5]. Bivalves use this state for defense. While closing and holding their shell valves shut, they avoid the high energy consumption that striated muscles would require for such an action. This enables them to remain closed for long time periods to escape potential threats. The catch mechanism is suggested to be protein-based [6,7,8,9], relying on two unique proteins within the adductor muscles: myorod (power-holding protein) and twitchin (catch-regulating protein). This conclusion stems from in vitro experiments using molecular contractile models. Experiments have demonstrated that these proteins can hold the contractile apparatus in the contracted state by cross-linking the thin and thick filaments of the contracted muscle under specific conditions [10,11]. However, the catch state research lacks critical studies, specifically in vivo and microscopic analyses, that could demonstrate how this state is established within living cells. Furthermore, few studies have addressed the supramolecular organization of the catch muscle contractile apparatus [12,13,14,15]. The formation, or myogenesis, of these muscles has only been sporadically investigated [16,17,18,19,20,21]. It is currently known that a temporary muscular system is formed and subsequently disintegrates during bivalve larval development, as the larva metamorphoses into a juvenile. This temporary muscular system consists primarily of striated rather than smooth muscles, a feature that likely reflects the evolutionary history of the molluskan muscular system. Interestingly, despite differences in muscle fiber organization, larval muscles express the same proteins as those found in smooth muscles of adult mollusks. For example, the expression of thick-filament contractile apparatus proteins, such as twitchin, myosin, and paramyosin, begins quite early [16], and their presence in the larval adductor and retractor muscles has been demonstrated by immunostaining [17]. However, the sequence of protein formation and their subsequent incorporation into myofilaments of the contractile apparatus remains unknown. Additionally, the role of myorod, a key protein involved in the cross-linking responsible for the catch state [9], in larval muscle formation has not yet been sufficiently clarified. This is explained by the challenge of distinguishing the molecule of myorod from that of molluskan myosin, because both are derived from alternative splicing of a single gene [22]. Until recently, it was also impossible to generate antibodies against the unique region of myorod, a protein that shares 85% of its molecular structure with myosin [23]. Both proteins share the same rod part and part of neck domain, but their head domains differ dramatically.

In the present study, we attempt to shed light on the roles of contractile proteins and their involvement in myofibril formation and spatial arrangement within the contractile apparatus of bivalve mollusks during their larval development. We also aim to elucidate whether the larval development of these mollusks can provide insight into the origin of their unique “catch” state. These aspects have remained largely unstudied until now. Attempts to investigate bivalve myogenesis were made previously as well. However, most of those studies focused on morphological description of the muscle system’s formation and relied mostly on staining of developing muscles with fluorescently labeled phalloidin, a filamentous actin marker. Some studies used antibodies against muscle proteins [17] to take a close look at how the larval muscle system forms and breaks down in mussels, *Mytilus trossulus*. However, those studies largely demonstrated the incorporation of thick-filament proteins into striated muscle myofibrils during the development of the muscle system, without fully elucidating the intrinsic mechanisms of the process. A deeper understanding requires the examination and comparison of the intermediate larval developmental stages. The species selected for our study, the oyster *Magallana gigas*, was also comprehensively analyzed previously [19] with confocal microscopy, which was successfully employed for phalloidin-based visualization of the larval muscle system’s development. However, the part of the study aimed at identifying contractile apparatus proteins other than actin during larval development proved to be less successful. This was due to the use of commercially available antibodies, originally raised against proteins from vertebrate muscles, to detect these contractile proteins. Indeed, the lack of commercially available antibodies against invertebrate proteins has posed a significant limitation for such research. In the present study, however, we successfully generate a series of specific antibodies against the proteins found in the thick and thin filaments of bivalves. The obtained antibodies are labeled twitchin, troponin C, myorod, and myosin molecules. These antibodies allow us to clarify how the major contractile proteins emerge and are incorporated into the contractile structures of mollusk’s larval muscles. Consequently, our study focuses on a detailed, step-by-step analysis of the muscle system’s development in *M. gigas*, a commercially valuable oyster species, using unique rabbit antibodies that we raised against isolated and purified bivalve antigens. No such studies have been undertaken previously.

## 2. Materials and Methods

### 2.1. Animals

Adult oysters (*M. gigas*) and mussels (*Crenomytilus grayanus*) (Mollusca: Bivalvia) were collected from Vostok Bay, Sea of Japan, near the Vostok Marine Biological Station, A.V. Zhirmunsky National Scientific Center of Marine Biology, Far Eastern Branch, Russian Academy of Sciences. Prior to the experiments, the animals were rinsed two to three times with filtered and UV-sterilized seawater. Spawning was induced by thermal shock, which involved lowering the temperature to 10 °C and then raising it to 18 °C over a period of 10–20 min. Spermatozoa and mature oocytes from five oysters were mixed in filtered seawater (with a salinity of 28‰) for fertilization at 17 °C. After registration of oocyte cleavage, the larval cultures were kept at 17 °C in 5 L beakers with constant agitation of the water column by an air jet directed at the water surface; the water was changed every two days. The stages of larval development were examined under a light microscope using criteria reported previously [24]. Samples were collected at the following developmental stages: unfertilized egg; blastula, 12 h post-fertilization (hpf); early, middle, and late trochophore (20, 24, and 28 hpf, respectively); early, middle, and late veliger (41, 72 hpf, and 5 days post-fertilization (dpf), respectively); and pediveliger (14 dpf). After 56 hpf, the larvae were fed microalgae, *Isochrysis galbana*, daily, at 100,000 cells/mL.

### 2.2. Preparation and Purification of the Muscle Protein Fractions

All of the proteins necessary for this study were previously isolated and further purified using the methods described for the mussel *C. grayanus* [25,26,27]. Twitchin and myorod were obtained via a protein isolation procedure involving dissociation of the contractile apparatus into thin and thick filaments. The target proteins were then separated using ammonium sulfate salting-out. Troponin C was prepared by a method involving the extraction of thin-filament surface proteins with a lithium chloride solution. After the isolation, twitchin and troponin C underwent fine purification via ion-exchange chromatography, as described previously [26,27]. The myorod was purified through cycles of depolymerization and clarification followed by polymerization and precipitation. Myorod preparation, via controlled proteolysis by a previously described method [28], was used to obtain its unique C-terminal domain. This domain, along with intact twitchin, myorod, and troponin C molecules, was used to produce the primary antibodies.

### 2.3. Production of Primary Antibodies Against Muscle Proteins

**Figure 1:**
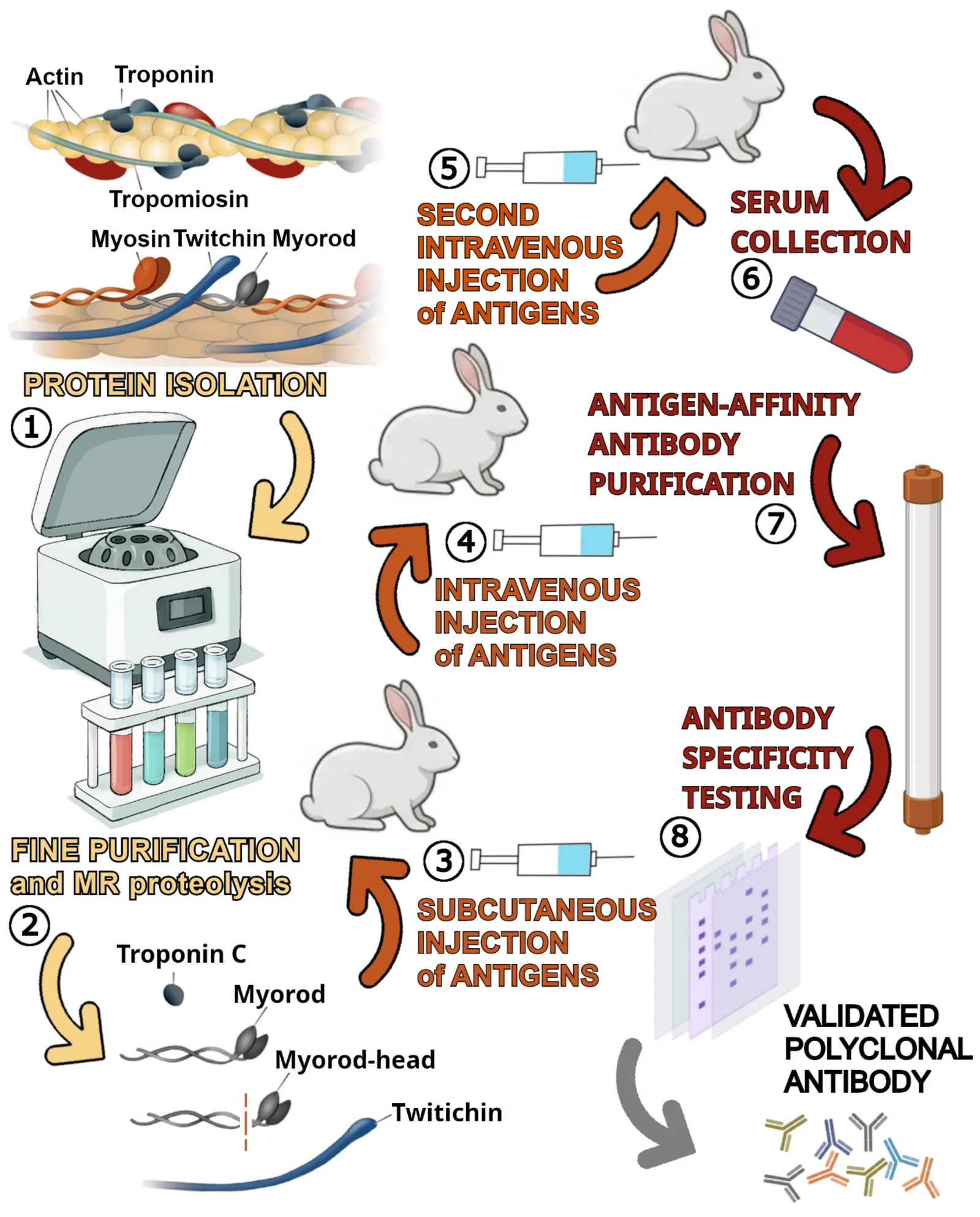
Production of polyclonal antibodies against muscle proteins from *C. grayanus*.

**Figure 2:**
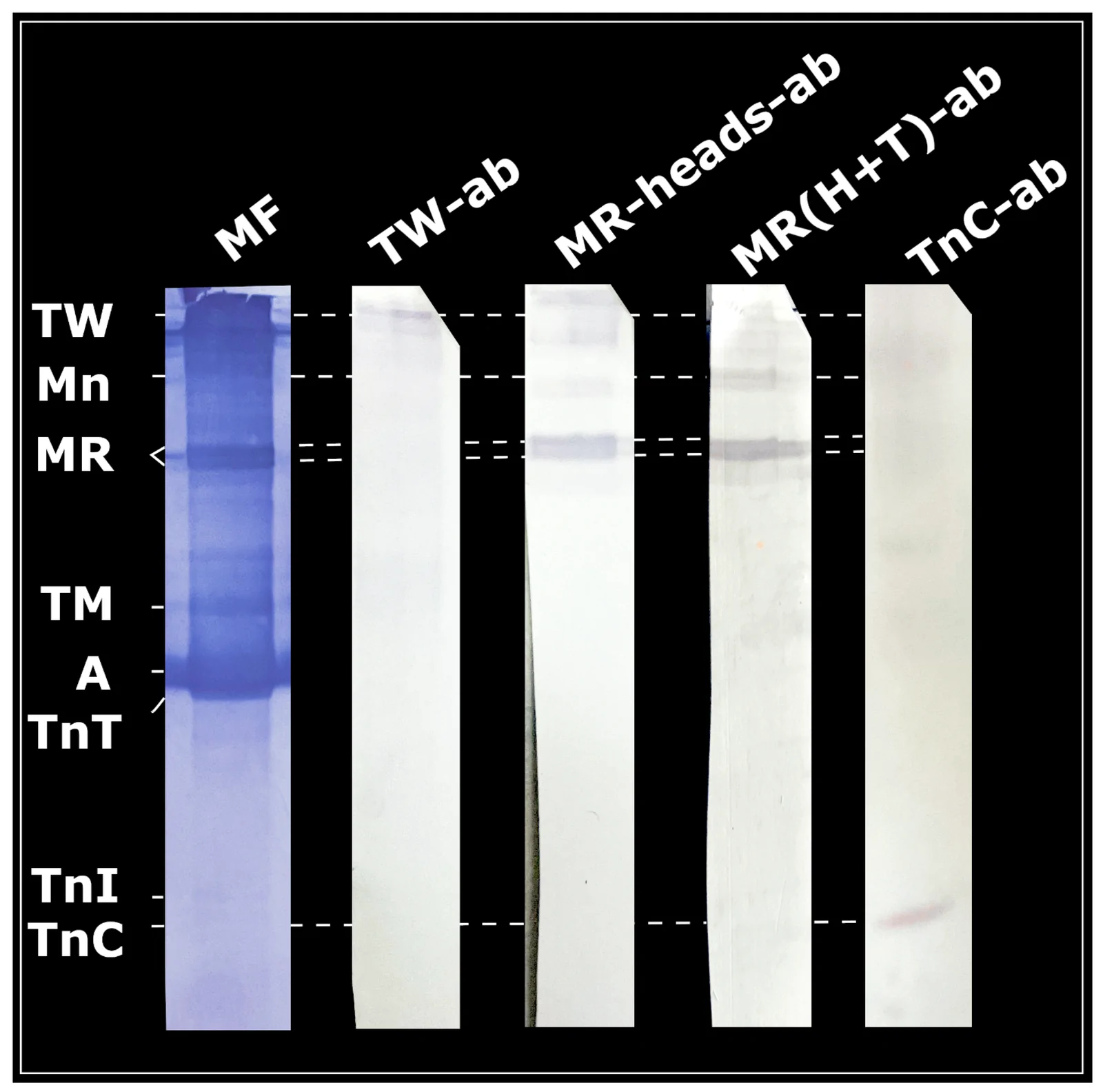
Specificity of primary mussel (*C. grayanus*) antibodies against oyster (*M. gigas*) muscle proteins. Antibodies are obtained against highly purified preparations of individual muscle proteins (twitchin, myorod, myorod head domain and troponin C) isolated from the mussel *C. grayanus.* Antibodies are tested on preparation of rigorized and washed oyster adductor homogenate (preparation content shown on the first lane, labeled “MF”). Abbreviations: MF—oyster “myofibrils”; TW—twitchin; Mn—myosin; MR—myorod; TM—tropomyosin; A—actin; TnT—troponin T; TnI—troponin I; TnC—troponin C; TW-ab—antibody against twitchin; MR-heads-ab—antibody against head domain of myorod; MR(H+T)-ab—antibody against full-length molecule of myorod (head and tail); and TnC-ab—antibody against troponin C.

To obtain the primary polyclonal rabbit antibodies against *C. grayanus* muscle proteins (myorod and myorod head domain, troponin C, and twitchin at a concentration of 100 mg/mL), a classic animal immunization protocol was used that included several stages [16]. First (Figure 1), the isolated and purified antigens, which were tested by the SDS-PAGE method [29,30], were emulsified with a full Freund’s adjuvant for the first subcutaneous injection and an incomplete set of Freund’s adjuvants for subsequent injections to enhance the immune response, and were administered intravenously. The emulsion was injected subcutaneously at several sites on each rabbit’s back with an insulin syringe. A total of 12 rabbits, three for each of the antigens, were used to produce the antibodies. The total injection volume was 1 mL per injection. Primary immunization was carried out at an interval of 2–4 weeks. Ten to 14 days after each injection, small blood samples were taken from the marginal vein of each rabbit’s ear to monitor the antibody titer using the Western blotting (WB) method with the previously detected antigens. When the antibody titer reached the desired level (usually 1:5000–1:10,000), a final blood sample was collected by cardiac puncture under anesthesia, after which the animal was euthanized in accordance with ethical standards. The resulting serum was separated, processed to remove nonspecific antibodies (e.g., by adsorption on immobilized proteins from other tissues), and then purified by affinity chromatography on a column with an immobilized antigen. The purified antibodies were tested for specificity by WB and immunohistochemistry to confirm the absence of unexpected cross-reactivity with other proteins present in the “myofibrils” preparation of oyster adductor (Figure 2). The data in Figure 2 (third lane) demonstrate the expected cross-reactivity of the antibody raised against a complete molecule of molluskan myorod with a myosin molecule. This is because the myorod and myosin molecules are 85% similar in terms of their amino acid sequences and share the same rod domain. Therefore, when analyzing the results of immunostaining, it should be taken into account that the most informative data regarding the distribution of these two proteins during larval development are obtained through a comparative analysis of staining with an antibody raised against the full myorod and its head domain. The other antibodies tested did not show any cross-reactivity with other molluskan proteins. For long-term storage, the antibodies were aliquoted and stored at −80 °C or in freeze-dried form at −20 °C.

The antigens for antibody production were extracted from the adductor muscles of Gray’s mussel (*C. grayanus*) using previously described biochemical methods for protein isolation (1). The extracted proteins (myorod, troponin C, and twitchin) were further purified using fine purification techniques. The unique C-terminal regions (or “heads”) of myorod were obtained by enzymatic digestion of myorod with papain (2). The myorod heads, intact myorod molecules, twitchin, and troponin C were injected subcutaneously into 12 rabbits (three rabbits per antigen), along with complete Freund’s adjuvant (3). After 2–4 weeks, sensitization was repeated, this time injecting the antigens intravenously in incomplete Freund’s adjuvant (4). This process was repeated after another 2–4-week period (5). Finally, blood serum containing antibodies was collected from the anesthetized rabbits (6). These antibodies were subsequently purified using affinity chromatography (7). The purified antibodies were tested for their specificity using Western blotting with the original antigens and a preparation of myofibrils from bivalves (8).

### 2.4. Immunochemistry

Unfertilized eggs and larvae at various developmental stages were fixed with 4% paraformaldehyde solution (Serva, Heidelberg, Germany) in 0.1 M phosphate-buffered saline (PBS), pH 7.4, for 2 h at 4 °C and washed three times with cold PBS. The larvae, starting from the veliger stage, were relaxed by gradually adding MgCl_2_ diluted in filtered sea water up to 7.5% prior to fixation. The samples were stored in PBS containing 0.03% NaN_3_ at 4 °C. To monitor changes in the developing muscular system using laser confocal microscopy, indirect immunofluorescence was employed with rabbit polyclonal antibody against the thick- and thin-filament proteins of adult mussel muscle (dilution 1:1000–5000), and with monoclonal mouse antibody against acetylated α-tubulin (Sigma, St. Louis, MO, USA; dilution 1:1000) or Alexa Fluor^®^ 555 Phalloidin (Invitrogen, Carlsbad, CA, USA; dilution 1:1000) as markers of filamentous cellular actin. The specificity of the antibodies against the proteins was verified by WB (Figure 2). Starting from the early veliger stage, samples were incubated in 5% EDTA for 60 min at room temperature to decalcify the larval shells prior to immunoreaction. This step was necessary to reduce the adhesion of antibodies to the shell and improved the quality of immunostaining [17,24]. To avoid nonspecific binding, the samples were incubated overnight at 4 °C in a blocking buffer containing 10% normal goat serum (Sigma, St. Louis, MO, USA), 0.25% bovine serum albumin (BSA), 0.5% Triton X-100, and 0.03% NaN_3_. The samples were incubated with primary antibodies in the blocking solution for 5 days, washed in PBS–Triton X-100 (five times for 10 min each) at 4 °C, and then incubated overnight at 4 °C in a mixture of goat anti-rabbit IgG antibody conjugated to Alexa Fluor 647 (Invitrogen, Carlsbad, CA, USA), and goat anti-mouse IgG antibody conjugated to Alexa Fluor 488 (Molecular Probes, Eugene, OR, USA), diluted 1:1000 in blocking buffer. Negative controls were obtained by omitting the primary antibody to demonstrate absence of specific staining with secondary antibody. Phalloidin was routinely added to the secondary antibody. The samples were then washed five times in PBS for 10 min, stained with 0.1 μg/mL DAPI (Sigma, St. Louis, MO, USA) in PBS to visualize the nuclei, washed again three times in PBS for 15 min, and finally embedded in glycerol. At each developmental stage (early, middle, and late trochophore; early, middle, and late veliger; pediveligers), at least 50 embryos were examined for each muscle protein antibody.

### 2.5. Analysis of Confocal Images

All samples were examined as whole-mount preparations under a Zeiss Celldiscoverer 7 confocal laser scanning microscope equipped with an Axiocam 506 (CCD, with pixel size 4.54 × 4.54 µm) (Carl Zeiss Microscopy GmbH, Jena, Germany). We used standard objectives, Plan-Apochromat 20× (NA 0.70) and 50× (NA 1.2 W), with an internal tube lens changer to expand the total system magnification range up to 100×. Excitation wavelengths were set up according to the manual for the ZEISS ZEN software (V. 3.2, blue edition, Carl Zeiss Microscopy GmbH, Jena, Germany): 385/30 for DAPI; 469/38 for AF488; 555/30 for phalloidin and 631/33 for AF647. The wide-field mode was used for a preview scan and the confocal mode to scan individual larvae. No specific procedures were used for projection. Identical acquisition and processing settings were used for comparisons of staining intensity. Image analysis was performed using Imaris (V. 9.0.1, Oxford Instruments, High Wycombe, UK). Three-dimensional (3D) images were reconstructed from Z-slices using ImageJ (V. 1.53c, National Institutes of Health, Bethesda, MD, USA). For each sample, a series of Z-slices was recorded at 1 µm intervals. The series of optical slices was also rendered as individual images and converted into the TIFF format. These images were then adjusted for contrast and brightness and combined into figures using GIMP (V. 3.2, GNU Project, Free Software Foundation, Boston, MA, USA).

## 3. Results

### 3.1. Antibody Characterization

Specificity of polyclonal antibodies against twitchin, the entire myorod molecule, the myorod head domain, and troponin C (Figure 2) was studied by the WB method as described earlier [27], using a preparation of washed “myofibrils” [31] from the adductor muscles of the oyster *M. gigas* (throughout this text, we are referring to a complex of rigorized thin and thick filaments isolated from a muscle homogenate, not to actual myofibrils, and, therefore, use the word “myofibrils” in quotation marks). An analysis of membranes after transfer and antibody staining revealed bands that corresponded to twitchin, myorod, myosin, and troponin C (Figure 2).

### 3.2. General Development of the Muscular System in Oyster Larvae

**Figure 3:**
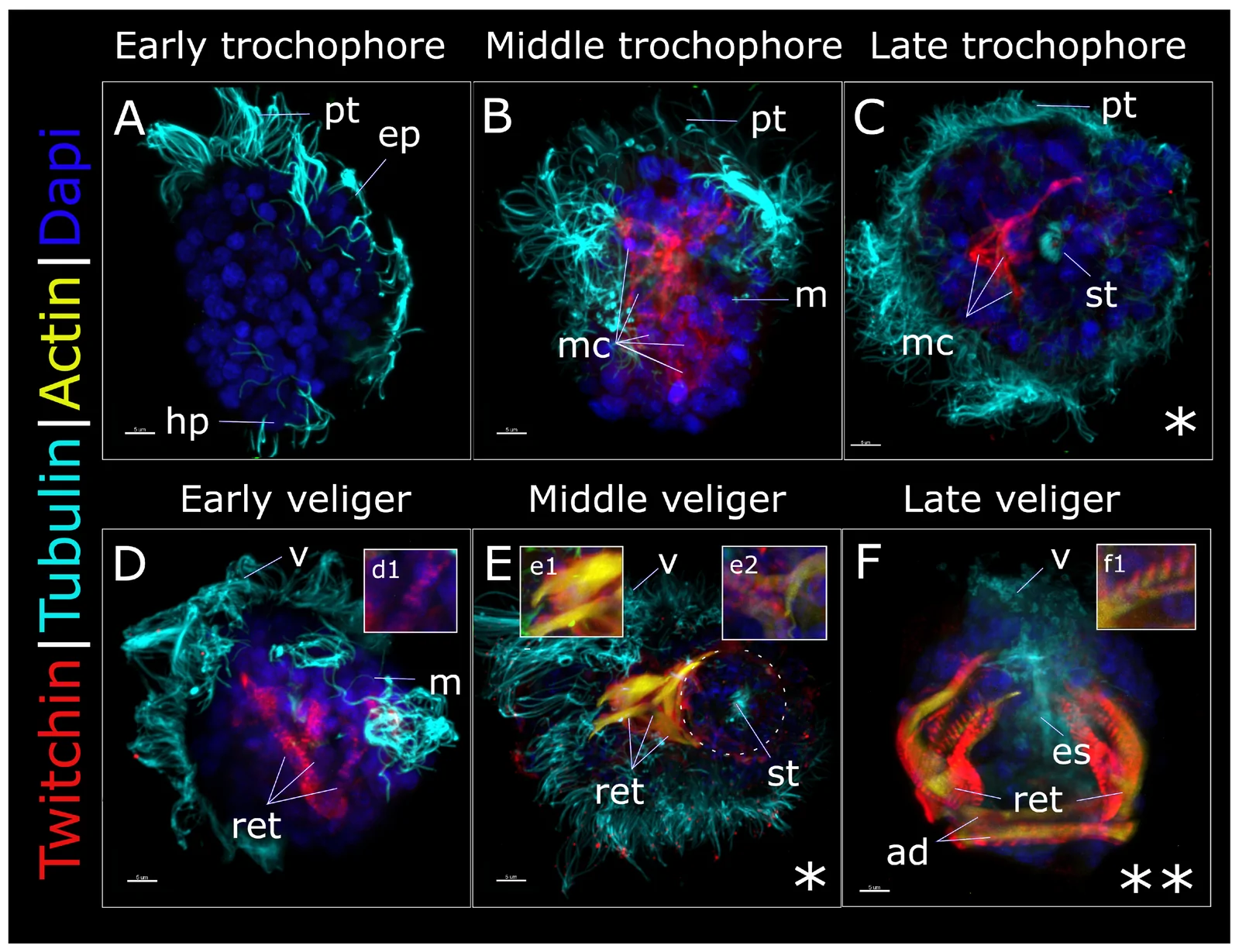
Twitchin, actin, and tubulin-like immunoreactivity in larval *M. gigas*. Larvae at different stages are labeled with anti-twitchin antibody (highlighted in red), anti-acetylated α-tubulin antibody (cilia) (cyan), phalloidin for detection of F-actin (yellow), and DAPI (nuclei) (blue). The apical pole is always upward, except in (**C**,**E**), which are views from the apical pole (indicated by an asterisk); the ventral side is on the right, except in (**F**), which is a view from the dorsal side (indicated by two asterisks). (**A**) Early trochophore (20 hpf) with ciliated cells. (**B**) Middle trochophore (24 hpf) with ciliated cells at the beginning of myogenesis; twitchin-immunopositive cells are designated as “mc”. (**C**) Late trochophore (28 hpf) with ciliated cells; twitchin-immunopositive cells are connected to each other, migrating toward the basal pole and forming the muscle ring (visible from the apical view, (**C**)). (**D**) Early veliger (41 hpf), with three pairs of well-defined velum retractor muscles and having a D-shape; a twitchin-associated striated pattern becomes visible (inset of (**D**), designated (**d1**)). (**E**) Middle veliger stage (72 hpf), with well-developed velum retractors that all have an obvious striated pattern for the thick-filament protein (inset of (**E**), designated (**e2**)). Actin appears in muscles, with its pattern, in contrast to that of twitchin, not striated (inset of (**E**), designated (**e1**)); the muscle ring can still be seen from the apical view ((**E**), outlined by circle). (**F**) In the late veliger stage (5 dpf), further differentiation of the muscle system occurs: branching of larval retractors becomes more profuse, the shell anterior adductor becomes visible, and the actin striated pattern becomes discernible (inset of (**F**), designated (**f1**)). Abbreviations: ad—anterior adductor; ep—episphere; es—esophagus; hp—hyposphere; m—mouth; mc—muscle protein-positive cells; pt—prototroch; ret—velum retractors; st—stomach; and v—velum. Scale bars = 5 μm.

**Figure 4:**
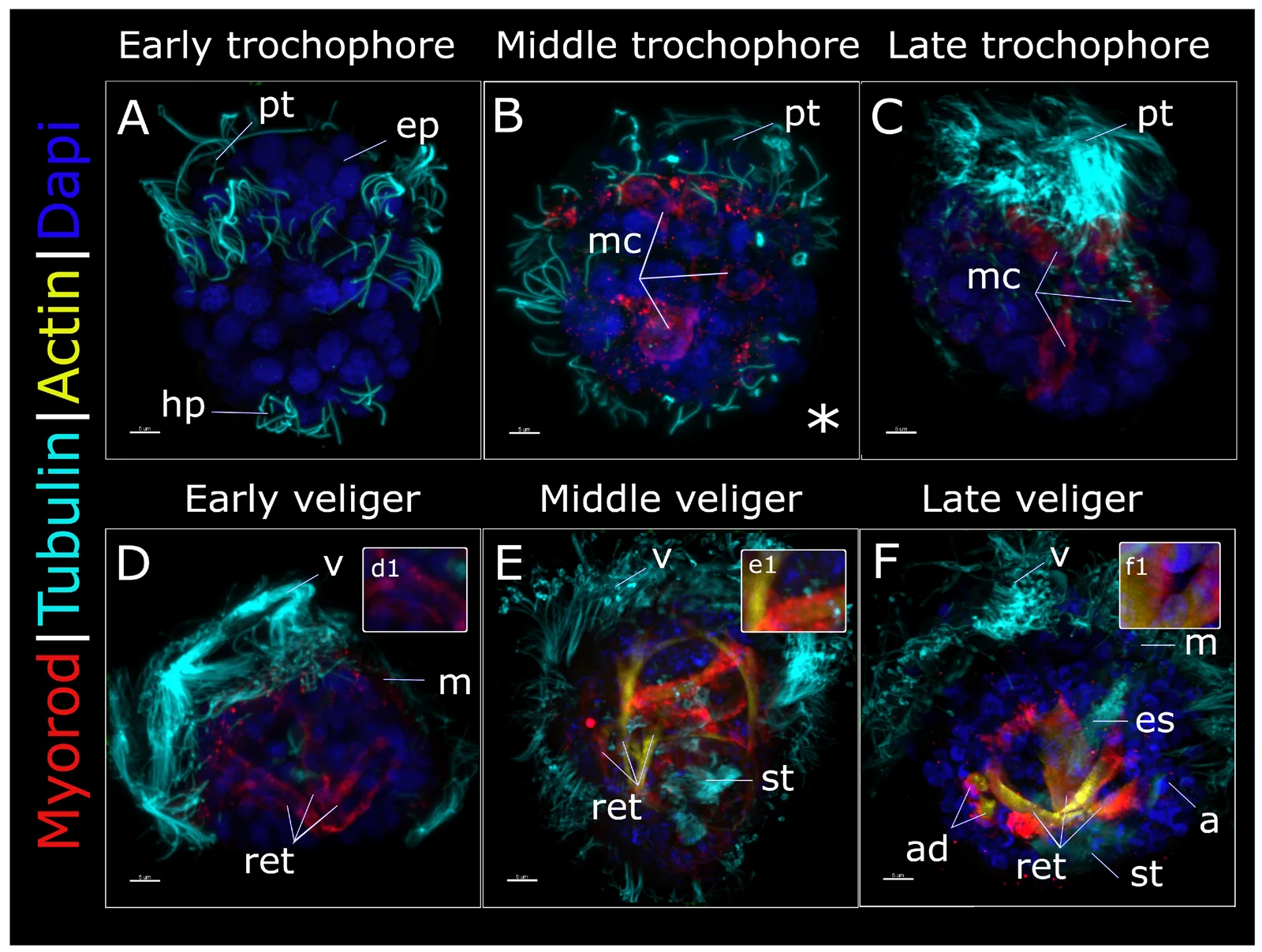
Myorod, actin, and tubulin-like immunoreactivity in larval *M. gigas*. Larvae at different stages are labeled with polyclonal anti-myorod-head antibody for detecting myorod molecules (highlighted in red), anti-acetylated α-tubulin antibody (cilia) (cyan), phalloidin for detection of F-actin (yellow), and DAPI (nuclei) (blue). The apical pole is always upward, except in (**B**), which is a view from the apical pole (indicated by an asterisk); the ventral side is on the right, except in (**A**), which is a view from the ventral side. (**A**) Early trochophore (20 hpf) with ciliated cells. (**B**) Middle trochophore (24 hpf) with ciliated cells at the beginning of myogenesis; myorod-immunopositive cells are designated as “mc”. (**C**) Late trochophore (28 hpf) with ciliated cells; myorod-immunopositive cells are connected to each other, migrating toward the basal pole. (**D**) Early veliger (41 hpf) with three well-defined pairs of velum retractors and having a D-shape; a myorod-associated striated pattern still cannot be seen (inset of (**D**), designated (**d1**)). (**E**) Middle veliger (72 hpf) with a changed shape and well-developed retractors; the thick-filament-associated striated pattern may be barely discernible (inset of (**E**), designated (**e1**)). Actin appears in muscles, with its pattern not striated either (inset of (**E**), designated (**e1**)). (**F**) In the late veliger stage (5 dpf), further differentiation of the muscle system occurs: branching of larval retractors becomes more profuse, the shell anterior adductor becomes visible, and both actin and myorod striated patterns become discernible (inset of (**F**), designated (**f1**)). Abbreviations: a—anus; ad—anterior adductor; ep—episphere; es—esophagus; hp—hyposphere; m—mouth; mc—muscle protein-positive cells; pt—prototroch; ret—velum retractors; st—stomach; and v—velum. Scale bars = 5 μm.

**Figure 5:**
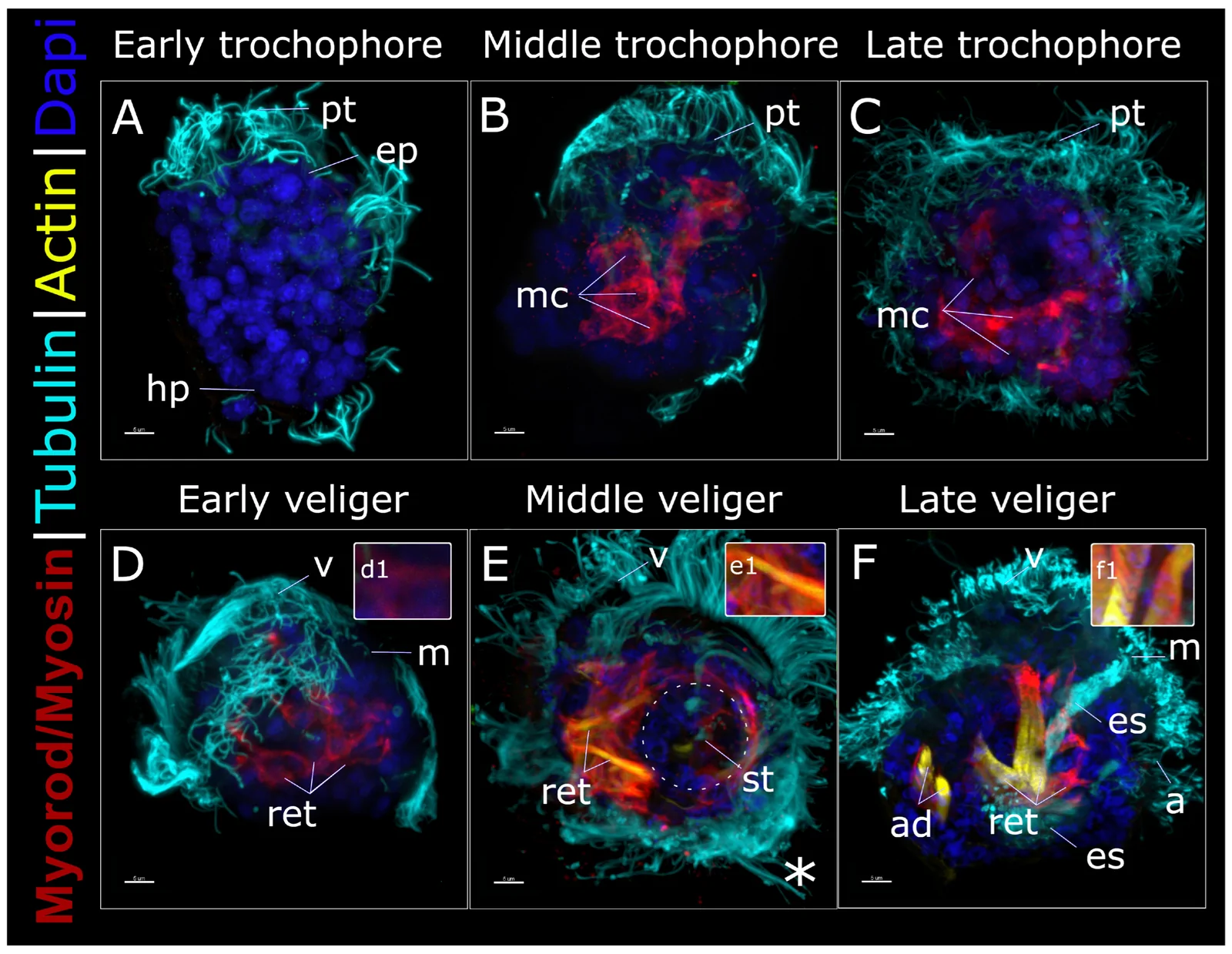
Myorod/myosin, actin, and tubulin-like immunoreactivity in larval *M. gigas*. Larvae at different stages are labeled with polyclonal anti-full-length myorod antibody also detecting myosin molecules (highlighted in red), anti-acetylated α-tubulin antibody (cilia) (cyan), phalloidin for detection of F-actin (yellow), and DAPI (nuclei) (blue). The apical pole is always upward, except in (**E**), which is a view from the apical pole (indicated by an asterisk); the ventral side is on the right. (**A**) Early trochophore (20 hpf) with ciliated cells. (**B**) Middle trochophore (24 hpf) with ciliated cells at the beginning of myogenesis. Myosin/myorod-immunopositive cells are designated as “mc”. (**C**) Late trochophore (28 hpf) with ciliated cells; myosin/myorod-immunopositive cells are connected to each other, migrating toward the basal pole and forming the muscle ring (visible from the apical view, (**E**)). (**D**) Early veliger (41 hpf) has three well-defined pairs of velum retractor muscles and a D-shape; a myosin/myorod-associated striated pattern still cannot be seen (inset of (**D**), designated as (**d1**)). (**E**) Middle veliger (72 hpf) with a changed shape and well-developed retractors that still lack the thick-filament-associated striated pattern (inset of (**E**), designated as (**e1**)). Actin appears in the muscles, with its pattern not striated either (inset of (**E**), designated as (**e1**)); the muscle ring can still be seen from the apical view ((**E**), outlined by circle). (**F**) In the late veliger stage (5 dpf), further differentiation of the muscle system occurs: branching of larval retractors becomes more profuse, the shell anterior adductor becomes visible, and both actin and myosin/myorod striated patterns become discernible (inset of (**F**), marked (**f1**)). Abbreviations: a—anus; ad—anterior adductor; ep—episphere; es—esophagus; hp—hyposphere; m—mouth; mc—muscle protein-positive cells; pt—prototroch; ret—velum retractors; st—stomach; and v—velum. Scale bars = 5 μm.

**Figure 6:**
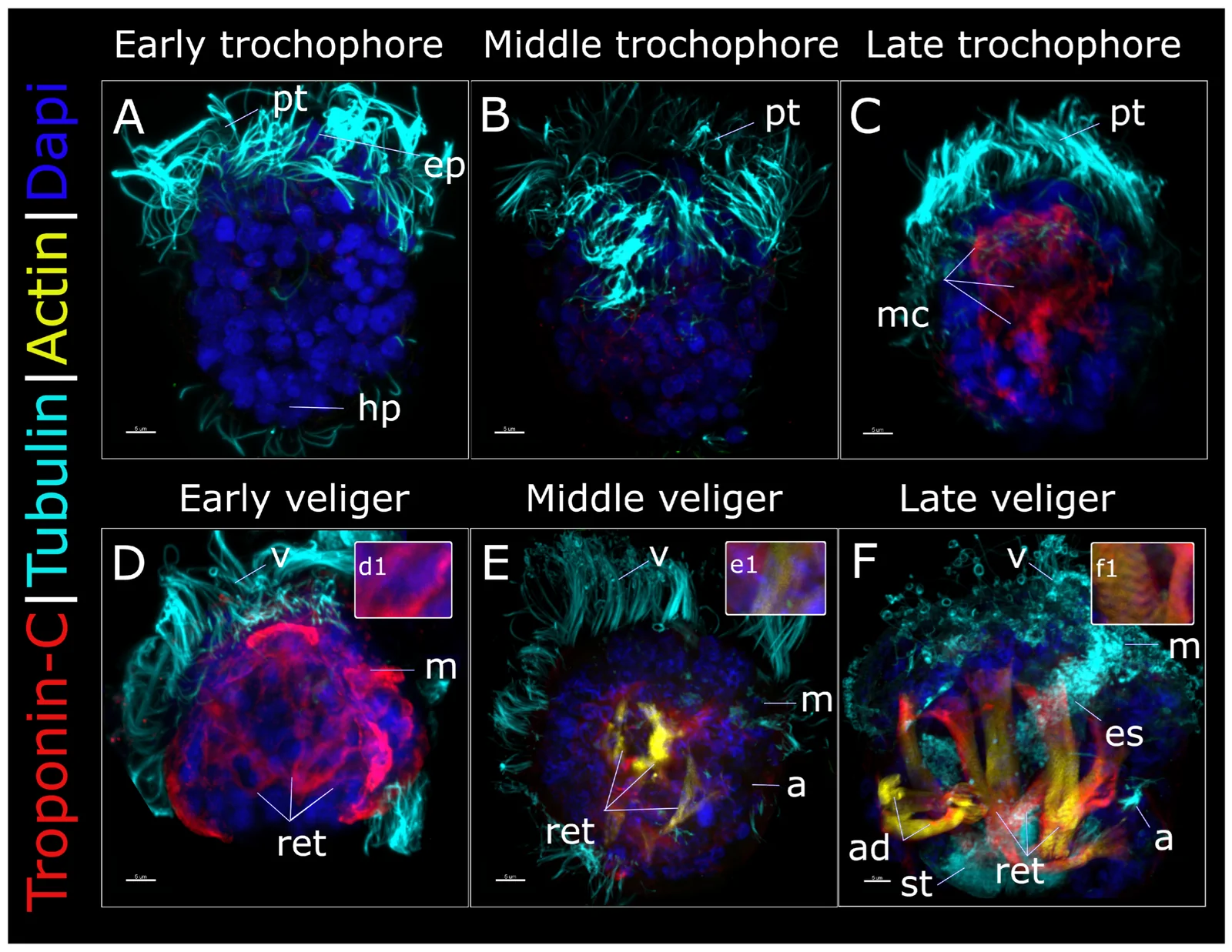
Troponin C, actin, and tubulin-like immunoreactivity in larval *M. gigas*. Larvae at different stages are labeled with anti-troponin antibody (highlighted in red), anti-acetylated α-tubulin antibody (cilia) (cyan), phalloidin for detection of F-actin (yellow), and DAPI (nuclei) (blue). The apical pole is always upward; the ventral side is on the right, except in (**A**), which is a view from the ventral side. (**A**) Early trochophore (20 hpf) with ciliated cells. (**B**) Middle trochophore (24 hpf) with ciliated cells. (**C**) Late trochophore (28 hpf) with ciliated cells at the beginning of myogenesis; troponin-immunoreactive cells are designated as “mc”. (**D**) Early veliger (41 hpf) with three developing pairs of velum retractors and having a D-shape; thin-filament-associated striated pattern still cannot be seen (inset of (**D**), marked (**d1**)). (**E**) Middle veliger (72 hpf) with a changed shape and well-developed retractors, containing actin, but still not striated (inset of (**E**), designated as (**e1**)). Actin appears in the muscles, and its pattern is not striated either (inset of (**E**), marked (**e1**)). (**F**) In the late veliger stage (5 dpf), further differentiation of the muscle system occurs: branching of larval retractors becomes more profuse, the shell anterior adductor becomes visible, and both actin- and troponin-associated striated patterns become discernible (inset of (**F**), designated as (**f1**)). Abbreviations: a—anus; ad—anterior adductor; ep—episphere; es—esophagus; hp—hyposphere; m—mouth; mc—muscle protein-positive cells; pt—prototroch; ret—velum retractors; st—stomach; and v—velum. Scale bars = 5 μm.

Using laser scanning confocal microscopy, we monitored the development of oyster larvae from the unfertilized egg stage through the pediveliger stage. However, the first evidence of immunopositive signals for muscle proteins was only recorded at the middle trochophore stage (24 hpf). Therefore, the data on the stages preceding the early trochophore stage (with negative muscle protein staining) are omitted. The latter stage, which occurred after 20 h, was identified by the larvae’s spheroidal shape, tapered slightly toward the bottom, and by the emergence of the ring of cilia (prototroch) that separates the episphere and hyposphere of the trochophore (Figure 3A, Figure 4A, Figure 5A and Figure 6A). At this stage, no signs of expression of the muscle proteins tested or the F-actin signal were observed. However, a few hours later, at the middle trochophore stage (24 hpf), some muscle proteins were detected by whole-mount antibody staining (Figure 3B, Figure 4B and Figure 5B). At this stage, cells with positive immunostaining for muscle proteins were visible. They clustered in a vertical orientation (Figure 3B and Figure 5B). By the late trochophore stage (28 hpf), the myoblasts had become elongated and appeared fused (which requires further investigation) to form muscle bundles (Figure 3C, Figure 4C and Figure 5C) that developed into striated velum retractors by the D-veliger stage (41 hpf) (Figure 3D and inset d1). From some angles of view (from the top, indicated by an asterisk in the figure), the velum retractors resembled a muscular ring surrounding the stomach (Figure 3C,E and Figure 4E).

**Figure 7:**
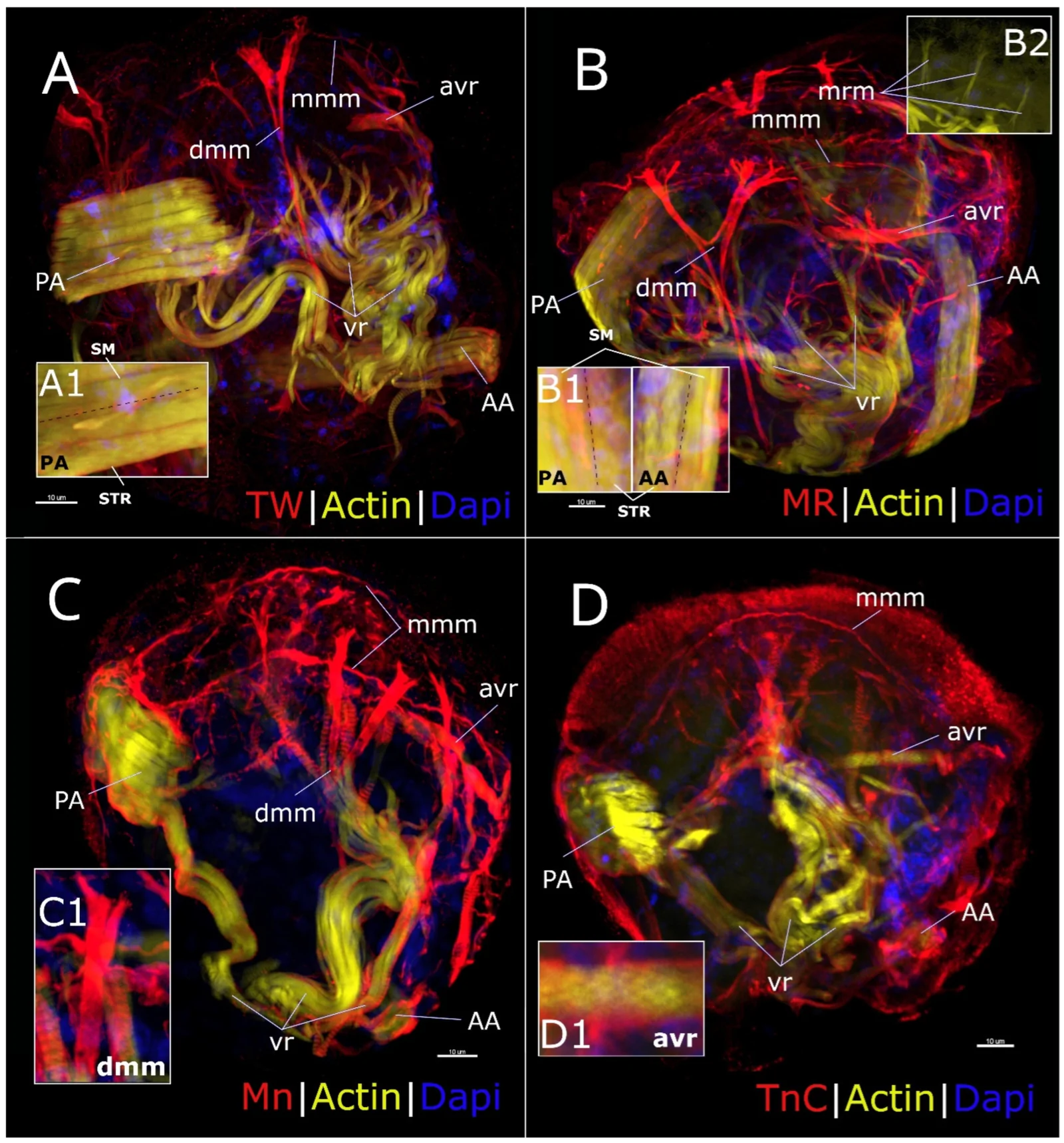
Lateral view of the major muscles of *M. gigas* pediveliger (14 days post-fertilization), with the ventral side at the top, the dorsal side at the bottom, the anterior side on the right, and the posterior side on the left. Larvae are labeled with antibodies against twitchin (**A**), myorod head domain (**B**), full myorod (detecting also myosin molecules) (**C**), or troponin C (**D**) (red); with phalloidin for detection of F-actin (yellow); and DAPI for detection of nuclei (blue). The specimen has a developing posterior adductor (PA), already exceeding the anterior adductor (AA) in size. In some parts, both adductors show signs of striation (inset (**A1**,**B1**); sm—smooth part; str—striated part; separated with dotted line). In addition, the specimen has long, branching, striated larval velum retractors (vr) and striated anterior ventral retractors (avr, inset (**D1**)), paired smooth mantle margin muscles (mmm), and bifurcating mantle retractor muscles (mrm, inset (**B1**)). The developing smooth digestive mass muscle (dmm, inset (**C1**)) can be seen (**A**–**C**) that still lacks actin and troponin labeling (and is, therefore, absent under letter (**D**)). Scale bars = 10 μm.

As mentioned above, the next stage of development was D-veliger. The larvae had a characteristic D-shape at this stage due to the formation of a shell at the basal pole (Figure 3D, Figure 4D, Figure 5D and Figure 6D). By this point, a clearly visible velum, the larvae’s locomotory organ, was fully formed at the apical pole. By 72 hpf (middle veliger stage), all three pairs of well-developed retractors were visible in the larvae (Figure 3E, Figure 4E, Figure 5E and Figure 6E). The retractors became distinctly striated only by the late veliger stage (5 dpf). The anterior adductor muscle was formed and clearly divided into two parts that visually lacked the striated pattern (Figure 4F, Figure 5F and Figure 6F, but not Figure 3F). The digestive system, which was also clearly visible, consisted of a stomach, a mouth, and anal openings. The posterior adductor that was partially striated (Figure 7A, inset A1; and Figure 7B, inset B1), the muscles that attached the mantle to the shell (mantle margin muscles), the mantle retractors (Figure 7B, inset B2), and the anterior ventral retractor muscles (Figure 7D, inset D1) emerged by the pediveliger stage (14 dpf) (Figure 7). The developing muscles of the digestive system and the digestive mass (Figure 7C, inset C1) were also visible.

### 3.3. Comparative Analysis of Muscle Protein Pattern in the Early Stages of Myogenesis in Oyster Larvae

Antibodies labeling the thick-filament proteins (myorod, myosin, and twitchin) could detect muscle cells as early as the middle trochophore stage, which occurred at 24 hpf (see Figure 3B, Figure 4B and Figure 5B). However, the emergence of muscle protein did not begin uniformly. While the staining for twitchin and the entire myorod molecule revealed clustered, elongated muscle cells forming a continuous band along the vertical axis of the larvae’s bodies at this stage (Figure 3B and Figure 5B), the staining with myorod-head-specific antibody detected this protein only in single, spatially separated cells (Figure 4B). In both cases, however, the proteins were found on the periphery of myoblasts. This data allowed us to conclude that the antibodies that bound to the entire myorod molecule at the middle trochophore stage, for the most part, stained myosin molecules. When discussing the identification of myosin in larval tissues below, we take into account the comparison between the antibodies against the entire myorod molecule (which stained both the myorod and myosin) and those against the head domain of myorod (which stained only the myorod).

Meanwhile, the staining to detect thin-filament proteins (actin and troponin) did not reveal any specific localization of these proteins in the larval tissues (Figure 6B). The staining with anti-troponin antibody showed a diffuse distribution of the protein. Phalloidin did not detect actin (Figure 3B, Figure 4B, Figure 5B and Figure 6B).

Significant changes in the myoblasts were observed during the next developmental stage (28 hpf). The staining of cells that contained thick-filament proteins revealed that twitchin and myosin, as well as myorod to a lesser extent, formed elongated structures that resembled the cords of future larval retractors (Figure 3C, Figure 4C and Figure 5C). During the late trochophore stage, these cells migrated toward the ventral side. When the larvae were viewed from the apical side, the emerging staining pattern resembled an open muscle ring (Figure 3C). A completely different pattern was observed when the larvae at this stage were stained with anti-troponin antibody (Figure 6C). In that case, a group of cells resembling the myoblasts that had expressed thick-filament proteins during the previous developmental stage was clearly visible in the central region of the larvae’s bodies. The staining of the larvae with fluorescently labeled phalloidin still yielded no results.

### 3.4. Myofibrillogensis and Myoanatomy of Veligers

By 41 hpf (D-veliger stage), the formation of larval retractors containing the major thick-filament proteins (myosin, myorod, and twitchin) had become evident (Figure 3D, Figure 4D and Figure 5D). Importantly, at this stage, only twitchin exhibited a distinct striated pattern and was, consequently, organized into sarcomeres (Figure 3D, inset d1). Structures resembling retractors could also be detected in the larvae stained with the antibody against troponin (Figure 6D). However, in addition to the retractors, the staining affected other cells located on the periphery of larval tissues. The staining of the larvae with phalloidin still did not reveal actin.

Actin was detected as early as the middle veliger stage (72 h). Nevertheless, the actin staining was uniform (see inset e1 in Figure 3E, Figure 4E, Figure 5E and Figure 6E) and contrasted with the already formed striated pattern of twitchin staining (see inset e2 in Figure 3E) and the forming striation pattern of myorod (Figure 4E and inset e1). At this stage, the troponin staining colocalized with the actin staining and became more confined to the retractor region (Figure 6E). Finally, at the late veliger stage (120 hpf), a well-defined striated pattern emerged in the organization of all the contractile proteins examined in this study that comprised the larval velum retractor structures (Figure 3F, Figure 4F, Figure 5F and Figure 6F). At the same time, a paired anterior adductor was formed, whose staining, however, did not reveal any striated pattern, except for the adductors stained for twitchin. In the latter case (Figure 3F), some widely spaced strips of staining could be seen. We probably recorded the very beginning of differentiation of the adductor structure into smooth and striated parts, as observed in adult animals.

The pediveliger stage (14 dpf) was characterized by a significant increase, more than threefold, in larval size from 40 to 150 μm (Figure 7). The muscles also increased in size simultaneously. The posterior adductor appeared partly striated (Figure 7, inset A1). Moreover, at this stage, the anterior adductor obviously demonstrated the same differentiation (Figure 7, inset B1). The posterior adductor became the largest muscle in diameter, and significantly exceeded both the velum retractors and the anterior adductor. The velum retractors, in turn, increased significantly in length, extending toward the velum and branching profusely. Closer to the margin of the mantle, the anterior ventral retractors could be observed. In some cases, the striation of these muscles was clearly visible (Figure 7D, inset D1). The long, narrow muscles of the mantle margin that run along the edge of the shell became noticeable. Perpendicular to these, there were the short, smooth mantle retractor muscles that branched at their ends (see Figure 7B, inset B2). Even though not clearly visible, both thick- and thin-filament muscle proteins could be detected in all of these muscles. However, a digestive mass muscle, characterized by the lack of actin and troponin labeling (and probably expression), could presumably be identified at this stage (Figure 7A–C). This muscle extended from the apex of the mollusk’s shell to the mantle and split into two large branches there. Notably, this muscle showed no signs of striation (Figure 7C, inset C1).

### 3.5. Structural Organization of Proteins in Striated Muscles of Oyster Larvae

**Figure 8:**
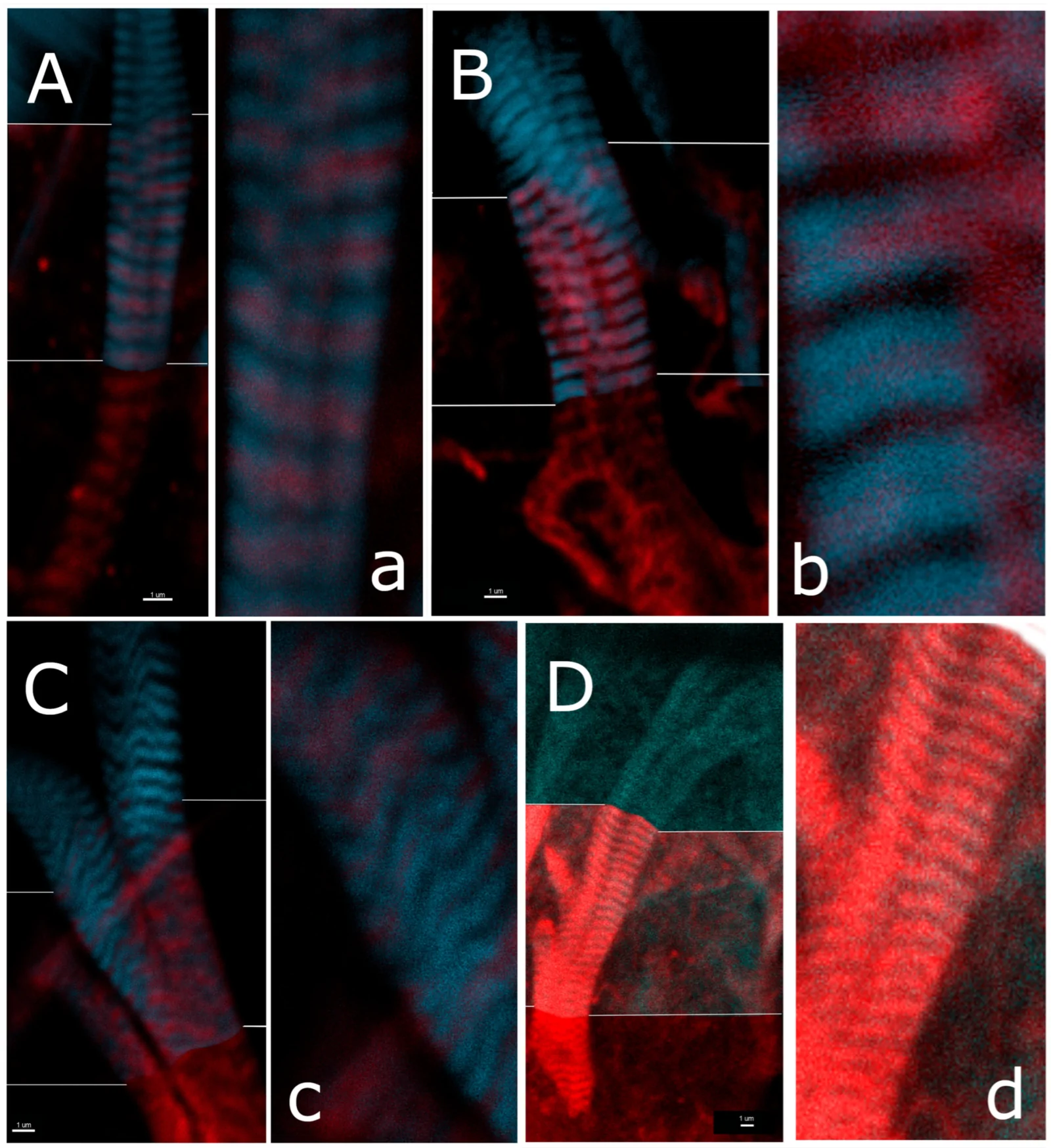
Striated pattern of velum retractors in larval *M. gigas*. Larvae are stained with antibodies against twitchin (**A**), myorod head domain (**B**), full myorod (to also detect myosin molecules) (**C**), or troponin C (**D**) (red); and with phalloidin for detection of F-actin (blue). Each image is divided into three parts: the top part shows the phalloidin staining; the bottom part shows the staining with antibodies against the muscle protein; and the middle part shows the overlap of both staining patterns. The right part of each image represents an enlarged view of the central area (designated with the same lowercase letter). The three images (**A**–**C**) are taken from a scan of the pediveliger stage (14 dpf), and the fourth image (**D**) is taken from the late veliger of *M. gigas* (5 dpf). Scale bars = 1 μm.

Examination of the structural organization of the pediveliger’s velum retractors (Figure 8A–C) under higher magnification revealed the thick-filament proteins (twitchin, myorod, and myosin) that formed a uniform striation pattern all along the muscle length (Figure 8A–C). The actin labeled with phalloidin formed double lines flanked by narrower zones that were stained positive for thick-filament proteins. These double lines were separated by slightly wider gaps devoid of muscle proteins. A comparison of the organization patterns of the thick-filament proteins showed that they were all localized in the same zones, as seen in the overlapping parts of Figure 8, and were surrounded on both sides by thin filaments. It was not surprising that the zones with thick and thin filaments overlapped slightly on the edges. However, unlike the double-line striation pattern in the pediveliger’s velum retractors, the actin and troponin in the veliger’s velum retractors formed a uniform striation pattern similar to that of the thick-filament proteins (Figure 8D). Furthermore, it was clearly evident that the zones occupied by actin and troponin overlapped completely. It was apparently that it could be more challenging to observe the double striation pattern due to the smaller size of the retractors at the veliger stage of development.

## 4. Discussion

In this study, we described the sequential stages of early myogenesis in *M. gigas*. For the first time, we provided a detailed, step-by-step description of the emergence and distribution of key muscle proteins in the contractile apparatus of bivalve muscles using primary antibodies against isolated bivalve antigens. Previous studies considered the major developmental stages of *M. trossulus* [16,17]. However, we analyzed the formation of larval muscles at higher temporal resolution. A comparable study on the same oyster species was undertaken in 2019 [19]. However, as the authors acknowledged, they did not manage to achieve high specificity in tissue staining due to the use of antibodies produced against rabbit proteins. Thanks to the synthesis of unique antibodies against bivalve muscle proteins, we identified the order in which the key muscle proteins become detectable during oyster embryogenesis, and tracked their organization into the contractile apparatus. Since phalloidin is commonly used to visualize actin, it is relevant to explain here why we generated antibodies against troponin. Phalloidin labeling does not provide reliable differentiation between the cytoskeletal and muscular isoforms of actin [17,32], and, for this reason, could not be used as a myogenic marker in our study. In contrast, antibodies against troponin C can be suitable for this purpose. Furthermore, these antibodies allowed us to observe, for the first time, the thin-filament assembly during larval development in bivalve mollusks. And, although its value as a specific myogenic marker requires further validation, in particular due to its diffuse early staining, we managed to record some signs of myogenesis and obtain new data, which, however, require further investigation.

### 4.1. Involvement of Thick-Filament Proteins in the Process of Larval Muscle Formation

According to the literature, muscle protein synthesis in bivalve larvae begins as early as the blastula stage (twitchin and myorod, 12 hpf) or the early trochophore stage (myosin, 17 hpf) [16]. However, we observed noticeable staining of the contractile apparatus proteins only at the middle trochophore stage (24 hpf). These data are consistent with previously published evidence indicating that the muscular system’s formation in mussels becomes detectable via immunostaining at approximately 30 hpf [17]. Nevertheless, the development of bivalve larvae depends heavily on rearing conditions, such as temperature, salinity, and feeding (for veligers). Therefore, in our study, we relied not only on time but also on the morphological characteristics of larvae [24]. Nevertheless, in our study, the first precursors of muscle cells containing significant amounts of myosin, myorod, and twitchin were observed to appear between 20 and 24 hpf. Most importantly, in all the larvae that were stained with antibodies against twitchin, the detected signal of the target protein was noticeably higher by 24 hpf. Twitchin clusters were observed in a greater number of cells, and its distribution was less diffuse than that of, e.g., myosin. Twitchin was primarily detected in the peripheral regions of the cells, clearly delineating their boundaries. A similar pattern was observed for full-length myorod antibodies (which also detect the presence of myosin). However, that distribution was more diffuse. Nevertheless, the staining with the antibody against the unique part of myorod (identifying only myorod) detected the protein in only four spatially separated myoblasts at that point. Consistent with earlier data on the late formation of thin filaments [17], the antibody against bivalve troponin produced barely noticeable staining at this stage. Although the order in which proteins are recognized by antibodies may be affected by factors such as the varying affinity of antibodies for target proteins, the availability of these targets (due to their interactions with other proteins), or the altered conformational structure of proteins themselves, we primarily evaluated the presence of proteins during development on the basis of their arrangement and distribution within the contractile apparatus. Furthermore, a sufficient affinity of antibody can be inferred from later stages of development, when both the structural patterns and intensity of protein staining are comparable. Thus, we suggest that we have determined the order of thick-filament protein emergence and accumulation in myoblasts: twitchin was the first protein detected in the forming muscle structure, followed by myosin, then myorod, and finally troponin (Figure 3, Figure 4, Figure 5 and Figure 6). This sequence could be traced through the contractile apparatus formation during the middle veliger stage. Twitchin was also the first to form distinct, elongated muscle bundles and the first to organize into filaments, exhibiting a striated pattern. A strikingly similar rapid development was observed during the formation of striations in vertebrate muscles, where the protein referred to as titin, belonging to the same family as twitchin [33], behaved in a similar way [34]. Importantly, the formation of the myorod-mediated muscle structure reached the detected level of myosin protein organization as early as the late trochophore stage. However, the striated distribution pattern of these proteins did not emerge until the late veliger stage, approximately 60 h after twitchin. In light of these findings, the previously advanced hypothesis that twitchin may serve as a molecular template for the assembly of the contractile apparatus in bivalve muscles is supported by compelling new evidence. This process strongly resembles that of skeletal muscle formation in vertebrates, where titin serves as a basis for the contractile apparatus assembly. Titin is known to bind to individual components of the sarcomere in vertebrate muscles. However, the ultrastructure of thick filaments and the localization of twitchin within the contractile apparatus in larval or adult bivalve muscles have not yet been sufficiently elucidated [35]. Thus, the role and position of twitchin in the contractile apparatus of bivalve muscles are still unclear. Therefore, the question remains open as to whether it, like titin, links the structures of the contractile apparatus. This assumption is supported by twitchin’s ability to interact with various muscle proteins, such as myosin [36], actin [37], paramyosin, and myorod [26]. In this study, we confirmed that twitchin staining is fully localized with myosin and myorod staining. However, this is only true for larval striated muscles where the sarcomere structure can be relatively easily observed. Nevertheless, the developmental plans for juvenile and adult bivalve muscles are likely fundamentally similar, because the striation in the developing adductors (at the late veliger stage) was first clearly visualized through immunostaining for twitchin (Figure 3F). Further study of smooth muscles in larval and juvenile bivalves is needed.

### 4.2. Involvement of Thin-Filament Proteins in the Process of Larval Muscle Formation

Although it is not the first report of a delay in the formation of thin filaments in bivalve muscles, we find, for the first time, that the labeling of surface regulatory proteins of thin filaments begins several dozen hours earlier than actin becomes detectable. Thus, troponin staining can be observed in larval retractors at 41 hpf, whereas actin incorporation into the retractors becomes noticeable by 72 h. Concurrently, the amount and distribution of actin suggest that the process of its incorporation into the filaments is not yet fully completed (striation is not yet discernible). These findings may indicate that the regulatory proteins of thin filaments emerge in the muscle tissues of larvae significantly earlier than actin itself. If this is indeed true, then the order of protein synthesis may serve to prevent myosin from binding to actin polymers while they are being synthesized. To avoid strong (rigorous) binding of actin and myosin during relaxation conditions, a pool of regulatory proteins should be prepared in advance. However, in view of the unique distribution of troponin throughout the larvae’s tissues during the late trochophore and early veliger stages, which differ from the distribution pattern of thick-filament proteins, it is possible that troponin is involved in signaling or cellular transport processes during the early stages of bivalve development. In this case, troponin would mediate the interaction of non-muscle proteins with actin. This study also shows, for the first time, that, due to the presence of troponin, thin filaments of bivalve larval muscles may be involved in the dual regulatory mechanism of bivalve muscles in the same way as in adult smooth muscles. Therefore, it is likely that muscle function in larval bivalves requires regulation of both thin (troponin) and thick (calcium-sensitive myosin) filaments [27,38,39]. Consequently, it cannot be ruled out that the calcium regulation of troponin may not be directly related to the regulation of the catch state of bivalve muscles [40]. However, this hypothesis is based on the assumption that the striated retractor muscles cannot enter the catch state in larval bivalves. Nevertheless, the results of our study indicate that all oyster muscles contain both twitchin and myorod during larval development. Therefore, they may be capable of the catch state. Thus, the catch state in bivalves may be the implementation of a function that emerged earlier as mollusks with striated muscles evolved rather than a trait acquired by their smooth muscles during evolution. However, these observations and hypotheses require further confirmation by genetic, biochemical, and physiological methods.

### 4.3. Structure of Larval Velum Retractor Sarcomere

The examination of the sarcomere structure in the retractor muscles of bivalve larvae yielded notable results. The first high-quality confocal images of these structures obtained can be compared to the images from a study of *M. trossulus* development [17]. In that study, a high level of detail was achieved by the Spatially Harmonically Improved Microscopy (SHIM) method rather than by confocal microscopy. The phalloidin-stained images we obtained (Figure 8A–C) most closely resemble the pattern observed in the study of striated muscles of adult scallops (Figure 4H in Dyachuk et al. [17]). However, the SHIM method visualized the distribution of myosin, not thin-filament proteins. On the other hand, we discerned a pattern consisting of two closely spaced lines separated by a wider one by visualizing muscle actin. Our results suggest that this pattern is formed by sequentially arranged sarcomeres separated by a wide gap, which appears to be a Z-line. This interpretation seems particularly plausible after visualizing the thick-filament proteins that are localized within the narrower gap of the pattern. Conversely, interpreting the paired convergence of zones from the pattern of myosin distribution in the scallop’s contractile apparatus is challenging because they contain myosin, not actin. Moreover, it is rather difficult to imagine a pattern similar to the one we discovered when examining the confocal scans in the above-cited article. The explanation is probably simple: we obtained most of the high-magnification scans by scanning retractor muscles at the pediveliger stage. There, the pattern can be clearly observed in low-magnification scans (Figure 8A,B). However, the pattern in such images is often hardly recognizable due to curvatures, tilt, or overlap of the muscles. Nevertheless, in the scans of veliger-stage larvae, which we used to visualize the colocalization of actin and troponin (Figure 6D), we observed a pattern similar to that in the retractors of veliger-stage mussels: the actin lines are separated by equal intervals.

Unfortunately, we cannot compare our phalloidin staining with that performed in a recent study of oyster development [19] because the images provided in that publication are of low resolution and lack high-magnification scans. The same situation is encountered in the article on *Yesso scallop* development [20], which also lacks high-magnification scans. However, a similar double-striation pattern can be seen in a large photograph of a scallop pediveliger. Since double staining (both for thick- and thin-filament proteins) was not used in that study, the pattern is difficult to identify. However, it can be found in the lower third of the velum retractors in Figure 2C of the published article by Sun et al. [20]. It is impossible, however, to examine the striated pattern of retractors in detail at earlier stages of the bivalve’s development due to the lack of photographs with sufficient magnification. Nevertheless, the pattern resembles those recorded for the oysters in our study and for mussels in previous studies. In addition, the authors, based on the results of electron and confocal microscopy [20], emphasize that the periodicities of the striations in the relaxed velum retractor muscles of the veliger and juvenile scallops are identical. Nevertheless, they do not provide a direct correlation between the zones. This is likely due to the lack of double staining of the muscles, which may raise questions about the broad dark bands between the sarcomeres of the pediveliger retractor muscles. It is also important to note that, when studying the sarcomere structure, we intentionally attempted to improve the quality of the muscle images, while other researchers likely did not. For example, we used specific scanning parameters, such as unidirectional laser movement and sequential scanning of each image layer across all channels. These parameters inherently require more time to take images. However, we did not apply them when taking general images of the larvae (Figure 3, Figure 4, Figure 5, Figure 6 and Figure 7). In those cases, we obtained images of sarcomeres that were unclear and misaligned, similarly to those reported in studies on development of other mollusks. These scans could not be used to accurately determine the relative positions of protein complexes. Their blurriness also prevented us from drawing conclusions about the distribution of thin and thick filaments within the sarcomere. Thus, in our view, the discrepancy between the patterns of thin- and thick-filament arrangement obtained through confocal microscopy and SHIM methods requires clarification specifically by the SHIM method. The difference between the patterns recorded by scanning of immunofluorescently stained larvae in our present study and those in an earlier study [17] is due to the structural differences in the contractile apparatus of bivalve larval muscles that depend on their developmental stage. Our comparison of the actin organization patterns of two stages, the veliger and pediveliger, confirms this. Our finding allows us to conclude that, although the muscles at the veliger stage resemble mature, striated muscles in appearance, they undergo further development as a bivalve reaches the adult stage. The causes and purposes of this development remain to be elucidated. However, such an elongation of sarcomeres without any increase in functional length may be associated with the need for rapid muscle elongation as a bivalve becomes an adult.

The last issue that requires in-depth discussion is the emergence of a muscle having no actin labeling and a noticeable decrease in the intensity of the actin staining at the distal ends of the larval retractors at the pediveliger stage. Both cases are characterized by a partial or complete lack of actin staining in the muscle. In our view, the underlying causes of these phenomena are the same. As previously reported by Dyachuk et al. [17], digestive mass muscles emerge at the pediveliger stage of bivalve embryogenesis. Since our two studies clearly demonstrate that muscle formation begins with the emergence of thick-filament proteins, followed by thin-filament proteins, it is very likely that actin has not yet formed thin filaments in these muscles by this time. This also explains the inability to detect these muscles with the antibody against troponin C. This may also explain the moderate labeling of actin in the distal portions of the retractors that branch and grow from the early veliger stage onward. Other studies have also reported a difference in the staining intensity between the distal and proximal regions of larval muscles [17,18,19,20].

In view of the fact that bivalve adductors are primarily characterized by a specific contractile state (catch state), as mentioned above, we would like to address the issue of their formation separately. As in a previous study on the Pacific oyster’s development [19], we observed the anterior adductor emerging at the veliger stage. However, in our study and in the article by Yurchenko et al. [24], the formation of the double anterior adductor followed retractor formation at approximately 72 hpf. In contrast, the study by Li et al. [19] on the oyster’s development reported that the adductor formed as early as in the D-veliger stage. However, the developmental timeline in that study was significantly earlier overall. Thus, the D-veliger stage occurred at around 24 hpf, in contrast to the 41 hpf recorded in our study and the 36 hpf reported by Yurchenko et al. [24]. Furthermore, the authors observed the late trochophore stage nearly twice as early (16 hpf vs. 28 hpf in our study). We assume this difference in the developmental rate is due to environmental conditions. Development in the study of Li et al. [19] took place at 20–23 °C, whereas, according to Yurchenko et al. [24], it occurred at 20 °C. In our study, the temperature was about 16–17 °C. Therefore, the difference in the order of muscle formation in the larvae turned out to be caused by temperature-induced acceleration of development. This prevented the observation of the intermediate stage, between the times of emergence of the retractors and the adductors, by Li et al. [19]. Overall, the issue of adductor formation requires further research, including by electron microscopy, due to the challenge of studying smooth muscle structure.

## 5. Conclusions

In our study, we managed to investigate the formation and development of the muscular system in oyster larvae, *M. gigas*, at the molecular level. We found that the leading protein in the contractile apparatus is twitchin. Using the immunoassay method, we detected this protein and found that it is the first protein to be incorporated into contractile structures and to form striations in larval striated muscles. The rest of the proteins in the thick filaments, myosin and myorod, demonstrate similar stages of involvement in the contractile apparatus, albeit with a significant delay. Actin molecules are the last to be detected in the contractile apparatus, which supports the literature data. This is of particular interest because the first signs of the presence of troponin C in muscle cells were detected dozens of hours earlier. The factor responsible for this this delay needs further investigation, and it may be related to the high affinity between myosin and actin in mollusks. Interestingly, in our study, troponin C was not the most effective indicator of the myogenesis process in mollusks, as its color was diffusely distributed in the early stages of larval development, bearing little resemblance to the emerging muscle structures. This suggests that troponin may be involved in other non-muscle processes that need further study. Nevertheless, it was a surprising finding that the protein involved in the calcium regulatory system of the contractile apparatus of smooth muscles in adult mollusks was detected in larval muscles. Additionally, the presence of myorod and twitchin proteins, which are responsible for the catch state of muscles in adult bivalves, was also found in larval striated retractors. This raises a question about the origin of the catch function in bivalve smooth muscles. The question of the functionality of the discovered proteins and structures remains open. It is not yet clear whether the larval striated muscles can enter the catch state, which could be addressed through physiological experiments on mollusk larvae. Additionally, ultramicroscopic studies are required to clarify if the structures formed by myorod and twitchin in the larval retractors match those of the smooth-muscle contractile apparatus in adult bivalves. The same approach could be used to answer the question of whether the narrow regions between the double actin bands identified in the study actually correspond to sarcomere Z-lines, and to monitor the process of sarcomere formation. In our work, we also found that the basic order of formation of larval striated muscles (retractors) and juvenile striated muscles (striated part of adductors) are similar. The first protein involved in the characteristic striation seems to be twitchin. A similar order—first the proteins of thick filaments, then the proteins of thin ones—also probably takes place in the development of smooth muscles, as seen in the example of digestive mass muscle. The distribution of proteins within individual muscles, the mechanism of accumulation of proteins in tissues without being incorporated into ordered structures, the synthesis of the contractile apparatus, and the consistency between the actual order of protein incorporation and the enzyme immunoassay data we have obtained are the issues to be addressed in the future, for which our work has paved the way.

## Data Availability

The authors confirm that the data supporting the findings of this study are available within the article. In addition, the raw data supporting these findings are available on request from the corresponding author.
